# Molecular epidemiology of a familial cluster of SARS-CoV-2 infection during lockdown period in Sant Kabir Nagar, Uttar Pradesh, India

**DOI:** 10.1017/S0950268821001989

**Published:** 2021-08-25

**Authors:** Kamran Zaman, Prem Shankar, Pragya D. Yadav, Dimpal A. Nyayanit, Sthita Pragnya Behera, Pooja Bhardwaj, Anita Shete, Triparna Majumdar, Rajaram Yadav, Savita Patil, Hirawati Deval, Gaurav Raj Dwivedi, Ashok K. Pandey, Rajeev Singh, Brij R. Misra, Niraj Kumar, Kaushik Kumar, Priyanka Yadav, Girijesh Kumar Yadav, Manoj Kumar, Kamlesh Kumar Sah, Ravi Shankar Singh, Sanjeev Kumar, Asif Kavathekar, Vijay Kumar, Rajni Kant

**Affiliations:** 1 ICMR-Regional Medical Research Centre, Gorakhpur, Uttar Pradesh, India; 2 ICMR-National Institute of Virology, Pune, Maharashtra, India

**Keywords:** Familial cluster, India, next-generation sequencing, SARS-CoV-2, Uttar Pradesh

## Abstract

We report a familial cluster of 24 individuals infected with severe acute respiratory syndrome-coronavirus-2 (SARS-CoV-2). The index case had a travel history and spent 24 days in the house before being tested and was asymptomatic. Physical overcrowding in the house provided a favourable environment for intra-cluster infection transmission. Restriction of movement of family members due to countrywide lockdown limited the spread in community. Among the infected, only four individuals developed symptoms. The complete genome sequences of SARS-CoV-2 was retrieved using next-generation sequencing from eight clinical samples which demonstrated a 99.99% similarity with reference to Wuhan strain and the phylogenetic analysis demonstrated a distinct cluster, lying in the B.6.6 pangolin lineage.

Severe acute respiratory syndrome-coronavirus-2 (SARS-CoV-2) was first reported from Wuhan, China [1]. Several studies have reported the transmission of SARS-CoV-2 from human to human, asymptomatic transmission and transmission in family and hospital settings [2, 3]. The first case in eastern Uttar Pradesh, India was reported from Basti town on 31 March 2020 [4]. Following this, the next case in this region was reported from Sant Kabir Nagar (SKN), a district adjacent to Basti [5]. The first case in SKN was reported on 15 April 2020 where a 71-year-old male who had visited New Delhi was found positive. The infection in this case was limited with no positive contacts. Following this a second case was noticed from SKN who was identified as the index case of the present cluster and he had returned home due to countrywide lockdown. Although he remained asymptomatic during his stay, the infection spread further leading to a family cluster. The present study describes in detail the asymptomatic transmission and delineates the transmission dynamics using next-generation sequencing (NGS).

## Study setting

A 23-year-old male student suspected to be infected with SARS-CoV-2 along with two of his co-travellers on the same bus returned to SKN from Deoband, Uttar Pradesh during the lockdown. He was tested positive for SARS-CoV-2 on 17 April 2020, following which he was quarantined. As part of contact tracing, all of his family members (28 individuals) and seven relatives who were residing in the same house were tested. In all the individuals, nasopharyngeal and nasal swab samples were collected by the state government health department and sent to our laboratory for testing. All the samples were processed as per the standard protocol and stored at −80 °C. Eighteen out of 35 members were found positive. The positive and negative tested family members were quarantined separately. On repeat testing of negative cases on 02 and 03 May 2020, five more members tested positive for SARS-CoV-2. Overall 12 out of 36 people in the house remain infection-free throughout their quarantine period (median quarantine period: 18 days, range 16–26 days). The secondary attack rate in this familial cluster was found to be 65.7% (assumption: all people got infected in the house before quarantine).

**Fig. 1.**
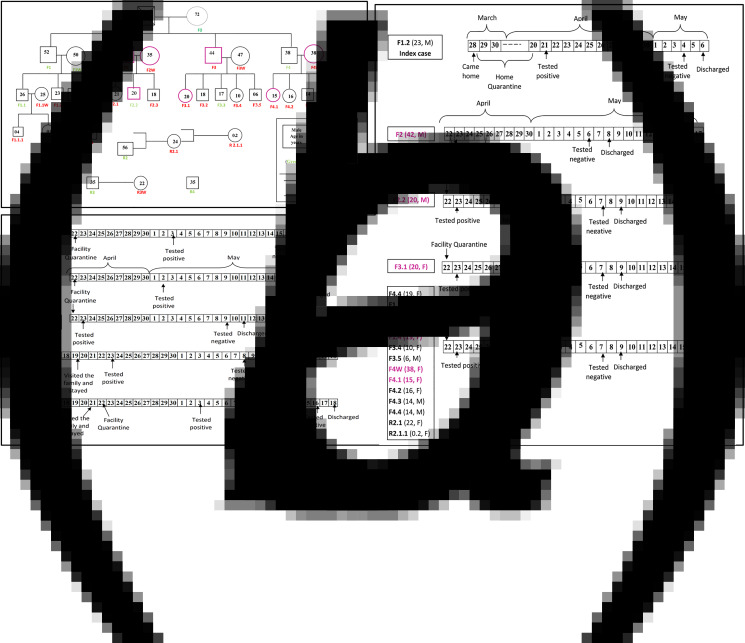
(a) Diagrammatic representation of family structure and their RT-PCR status for SARS-CoV-2 infection. (b) Chronological events with symptoms, date and duration of symptoms in each infected individual. *In the figure letter ‘F’ denotes family member, the letter ‘R’ denotes relative and the letter ‘W’ means wife. Numbers after letter represent generation separated by a dot (.) and chronological order among siblings; for example, if code is F2 this means a family member of the first-generation and second among siblings according to chronological order. Code F2W means the wife of F2. Similarly, F1.1.1 means the first member of the third generation of F1. We have denoted the only survivor of the oldest generation in the family as F0.

The median age in this cluster was 20.5 years (range: 2 months−72 years). The cluster had 10 family members below 18 years of age. This cluster was composed of 19 males (52.8%) and 17 females (47.2%). To obtain the ‘*P* value’ for the association, Fisher's exact test was applied (Table 1). The family lived in a pukka (cemented) house with a total of eight living rooms and a separate kitchen. Overcrowding was noted in the family. Among the individuals tested positives, only three out of 24 developed mild symptoms. Details of the chronological events and family structure are shown in (Fig. 1). Three members in this cluster had co-morbid conditions and were also found infected. All 24 infected individuals recovered from infection and no case fatality occurred in this cluster.

**Table 1.** Personal and clinical characteristics of the familial cluster in Sant Kabir Nagar, Uttar Pradesh, India

Characteristics		Frequency (column %)		
		Infected, *n* = 24	Non-infected, *n* = 12	*P* value (Fisher's exact test)
**Age in years**	⩽9	4(16.7)	1(8.3)	0.37
	10–19	8(33.3)	2(16.7)	
	20–39	9(37.5)	5(41.7)	
	⩾40	3(12.5)	4(33.3)	
**Gender**	Female	15(62.5)	2(15.4)	**<0.01**
	Male	9(37.5)	10(84.6)	
**Marital status**	Married	6(25.0)	5(41.7)	0.10
	Unmarried	18(75.0)	6(50.0)	
	Widowed	0(0.0)	1(8.3)	
**Primary occupation**	Unemployed	5(20.8)	2(16.7)	0.75
	Housewife	3(12.5)	3(25.0)	
	Student	13(54.2)	4(33.3)	
	Labourer	2(8.3)	2(16.7)	
	Other	1(4.2)	1(8.3)	
**Symptoms**	Symptomatic	3(12.5)	1(8.3)	0.65
	Asymptomatic	21(87.5)	11(91.7)	
**Co-morbid illnesses**	Present	3(12.5)	0(0.0)	0.54
	Absent	21(87.5)	12(100.0)	

## Next-generation sequencing

**Fig. 2.**
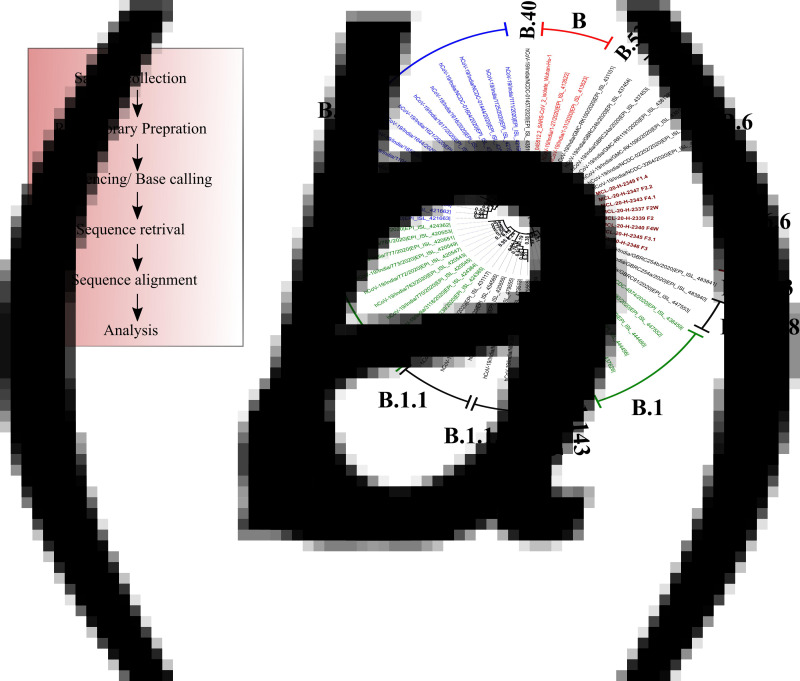
Phylogenetic tree of the coding region of the SARS-CoV-2. *A neighbour-joining tree of the SARS-CoV-2 sequences retrieved from the clinical samples. The tree was constructed using the representative GISAID clade sequences with the Tamura−Nei parameter model with a gamma distribution as the rate parameter. The bootstrap replication of 500 cycles was used to assess the statistical robustness of the generated tree. The scale depicts the number of base substitutions per site. The tree is coloured as per the GISAID clade and taxa. Taxa colour: (blue: B.4; red: B and green: B.1). Sequences retrieved in this study are marked in brown colour and are bold faced.

NGS was conducted by preparing RNA libraries from the extracted RNA. The RNA libraries prepared were sequenced using the Illumina platform (Qiagen, Germany) to delineate the transmission dynamics using the positive samples to retrieve the complete genomic sequence of the SARS-CoV-2. The detailed protocol of the method used is described elsewhere [6]. The pipeline used to obtain the SARS-CoV-2 sequences is depicted in Figure 2a. The retrieved sequences were aligned using the representative GISAID Indian SARS-CoV-2 sequences. A neighbour-joining tree was generated using the best model in MEGA version 7.0 [7]. A bootstrap replication of 1000 replication was used to assess the statistical robustness. Amino acid variations were also observed for the different proteins encoded by SARS-CoV-2.

The percentage of genome recovered from 24 samples ranged from 1.49 to 99.99 and the relevant reads mapped lay between 0.0 and 92.35%. The details of the percentage of relevant reads mapped and the percentage of the genome retrieved for all the 24 samples are tabulated in Table 2. Eight genomic sequences were retrieved with a genome coverage ranging between 99.94 and 99.96% while the other 16 sequences were below 95.5%. The neighbour-joining tree as generated using the Kimura-2-parameter model demonstrated that the retrieved sequences lay in the B.6.6 pangolin lineage (https://pangolin.cog-uk.io/). Two distinct clusters were observed for the generated tree, one with the B pangolin lineage variants comprising of B.B.4, B.6, B.6.6 lineages and other with B.1 pangolin variants (B.1, B.1.1.306, B.1.36.8) (Figure 2). The amino acid variation analysis for different structural and non-structural proteins demonstrated the presence of multiple variant amino acid sites in the ORF1ab at position nsp2(G519S), nsp3(P1010S,T2016 K), nsp4(N2767 T) nsp6(L3606F), nsp12(A4489 V), nsp13(G5411 V), whereas protein S, ORF3a and ORF8 showed no distinct variation (Technical Appendix Table 1). The amino acid position of ORF1ab protein L3606F (nsp6) is shared with clade O and A3i, and substitution at position A4489 V (nsp12) is shared with clade A3i. The nucleocapsid protein of the studied strain showed a single variable site in amino acid sequence at position P9265L. The percentage of nucleotide and amino acid similarity for different genes are tabulated in the Technical Appendix Table 2.

**Table 2.** Details of the samples collected and the RT-PCR value for different genes of the 24 clinical samples tested along with the percentage of relevant reads mapped and percentage of genome retrieved

Sr no.	MCL no.	Person ID	Age	Gender	E gene	ORF gene	S gene	Total reads	% of relevant reads	% of genome retrieved
1	MCL-20-H-2336	F1.2	23	M	34	36	37	1 690 508	0.000769	4.330669
2	MCL-20-H-2328	F3.4	10	F	32	30	30	1 507 078	0.00292	9.841822
3	MCL-20-H-2329	F3.2	18	M	23	24	22	2 999 722	0.002767	15.77768
4	MCL-20-H-2330	F1.1.1	4	M	22	24	24	1 324 178	0.05309	78.99542
5	MCL-20-H-2331	R2.1.1	0.2	F	32	31	31	1 168 428	0.000599	1.498177
6	MCL-20-H-2332	F4.5	8	F	36	34	34	730 536	0.031073	46.83811
7	MCL-20-H-2333	F3.5	6	F	33	31	31	1 198 500	0.083605	85.89105
8	MCL-20-H-2334	R2.1	22	F	34	32	33	1 184 166	0.009205	26.45554
9	MCL-20-H-2335	R3W	22	F	17	17	15	1 826 444	0.027923	67.2976
**10**	**MCL-20-H-2337**	**F2W**	**35**	**F**	**25**	**24**	**24**	**1 382 850**	**11.18733**	**99.94984**
11	MCL-20-H-2327	F2.1	21	F	27	28	26	1 123 962	0.310153	98.70582
12	MCL-20-H-2338	F1.1W	25	F	34	31	31	3 079 116	0.038095	76.32345
**13**	**MCL-20-H-2339**	**F2**	**42**	**M**	**21**	**19**	**19**	**5 830 376**	**72.60216**	**99.99666**
**14**	**MCL-20-H-2340**	**F4W**	**38**	**F**	**17**	**16**	**15**	**1 186 078**	**92.3591**	**99.95987**
15	MCL-20-H-2341	F4.2	16	F	34	33	32	1 080 084	0.233315	94.26813
16	MCL-20-H-2342	R1	15	M	33	31	32	1 814 786	0.052844	74.56442
**17**	**MCL-20-H-2343**	**F4.1**	**15**	**F**	**21**	**18**	**19**	**3 114 826**	**10.5289**	**99.95653**
18	MCL-20-H-2344	F4.4	14	M	34	32	32	1 259 566	0.028026	37.8758
**19**	**MCL-20-H-2345**	**F3.1**	**20**	**F**	**19**	**17**	**17**	**1 963 880**	**28.5716**	**99.95653**
**20**	**MCL-20-H-2346**	**F3**	**44**	**M**	**18**	**17**	**16**	**1 069 632**	**60.88253**	**99.95653**
**21**	**MCL-20-H-2347**	**F2.2**	**20**	**M**	**22**	**20**	**20**	**7 057 582**	**6.97756**	**99.95653**
22	MCL-20-H-2348	F4.3	14	M	26	26	25	1 724 272	0.20281	97.61228
**23**	**MCL-20-H-2349**	**F1.4**	**19**	**F**	**23**	**22**	**21**	**4 528 836**	**17.57688**	**99.96321**
24	MCL-20-H-2350	F3W	47	F	34	32	32	1 688 878	0.147909	95.29144

Bold values in the table 2 reflect the eight clinical samples in whom the genomic sequences were retrieved by NGS and used in the phylogenetic analysis.

## Discussion

We describe here a familial cluster (*n* = 24), out of 36 who were found to be infected with SARS-CoV-2. The index case had a travel history and spent 24 days in the house before being tested and was asymptomatic. Physical overcrowding in the house provided a favourable environment for intra-cluster infection transmission. Restriction of movement of family members due to countrywide lockdown limited the spread in the community. Most of the infected individuals were asymptomatic and those having symptoms had only mild ones.

Of the negative family members, five turned positive on repeat sampling. The possibility of asymptomatic and reverse transcription-polymerase chain reaction (RT-PCR) negative individuals transmitting the infection has been shown in several studies with possible reasons for false-negative results of RT-PCR due to insufficient viral specimens and the low load of virus in the upper respiratory tract infection [8]. The viral load in symptomatic and asymptomatic individuals is shown to be similar [9] and they serve as potential source of infection in the community and hospital settings [3, 10].

Females were infected more than males. Other factors like age, occupation, co-morbidity and marital status were not found statistically significant. The present cluster had 10 members of age <18 years, of which two were <5 years. Children being infected from the family members given the proximity and asymptomatic nature of the illness has been documented [8].

In India, multiple SARS-CoV-2 clades are reported to be circulating. The phylogenetic analysis demonstrated a distinct cluster, lying in the B.6.6 pangolin lineage. Further, genetic analysis of the sequences in this study demonstrated conserved amino acid variation in the ORF1ab regions. The variations were observed in the amino acid that contains trans-membrane domain (nsp3, nsp4 and nsp6), RdRp (nsp12) and helicase (nsp13). The implications of these changes need to be further explored.

This study provides insight into transmission of SARS-CoV-2 within a crowded household setting using genome sequencing, which is of notable value when assessing cluster outbreaks and transmission dynamics. With novel variants requiring attention and resources, the window period for such cluster analysis should be considered timely. However, of the 24 samples, genome was retrieved in only eight samples which might be due to low viral load; this highlights the limitation of sequencing in such situations.

## Conclusion

This study encourages future implementation of genome sequencing when addressing outbreaks in real-time, particularly in crowded household settings, and tangentially highlights the effectiveness of lockdown measures.

## Data Availability

All data generated and/or analysed during the current study are available from the corresponding author on reasonable request.

## Supplementary material

For supplementary material accompanying this paper visit http://dx.doi.org/10.1017/S0950268821001989. click here to view supplementary material
