# Supplementary material for: Molecular epidemiology of a familial cluster of SARS-CoV-2 infection during lockdown period in Sant Kabir Nagar, Uttar Pradesh, India
Source: Epidemiol Infect. 2021 Aug 25;149:e200. doi: 10.1017/S0950268821001989 (PMC8438426; doi:10.1017/S0950268821001989)
Supplement: Supplementary file 1 [file hygsup.zip › S0950268821001989sup001.docx]

Technical Appendix Table 1: *Changes in the amino acids of the SARS-CoV-2 sequences retrieved from the clinical samples of Sant Kabir Nagar, UP along with the representative sequences from different GISAID clade with respect to the SARS-CoV-2 isolate Wuhan-Hu-1 (Accession number NC_045512.2). GISAID Clades are depicted in different colours: Orange- L; Light Green- S; Dark Green- V; Light Blue-G; Dark Blue-GH; Light Red –GR; Grey-O. Purple- A3i*

| **NC 045512.2 SARS-CoV-2 isolate Wuhan-Hu-1** | **ORF1ab** | | | | | | | | | | | | | | **S** | **ORF3a** | | **ORF8** | **N** | | | | **Pangolin Lineage** |
| --- | --- | --- | --- | --- | --- | --- | --- | --- | --- | --- | --- | --- | --- | --- | --- | --- | --- | --- | --- | --- | --- | --- | --- |
|  | **207**  **(nsp2)** | **378**  **(nsp2)** | **519**  **(nsp2)** | **1010 (nsp3)** | **1515 (nsp3)** | **2015 (nsp3)** | **2016 (nsp3)** | **2767 (nsp4)** | **2796 (nsp4)** | **3606 (nsp6)** | **4489 (nsp12)** | **4532**  **(nsp12)** | **4715**  **(nsp12)** | **5411 (nsp13)** | **7711** | **8428** | **8622** | **9214** | **9265** | **9446** | **9455** | **9456** |  |
|  | **R** | **V** | G | P | **S** | S | **T** | N | **M** | **L** | **A** | D | **P** | G | **D** | **Q** | **G** | **L** | **P** | S | **R** | **G** |  |
| hCoV-19/India/GMC-RK100/2020\|EPI ISL 431101\| | . | . | . | . | . | . | . | . | . | . | . | . | . | . | . | . | . | . | . | . | . | . | B.53 |
| hCoV-19/India/1-27/2020\|EPI ISL 413522\| | . | . | . | . | . | . | . | . | . | . | . | . | . | . | . | . | . | . | . | . | . | . | B |
| hCoV-19/India/1-31/2020\|EPI ISL 413523\| | . | . | . | . | . | . | . | . | . | . | . | . | . | . | . | . | . | S | . | . | . | . | B |
| hCoV-19/India/NCDC-01457/2020\|EPI ISL 435108\| | . | . | . | . | . | . | . | . | . | F | . | . | . | . | . | . | V | . | . | . | . | . | B.40 |
| hCoV-19/India/GBRC9/2020\|EPI ISL 435056\| | . | . | . | . | . | . | . | . | . | . | . | . | L | . | G | . | . | . | . | . | . | . | B.1.145 |
| hCoV-19/India/GBRC113/2020\|EPI ISL 451158\| | . | . | . | . | . | . | . | . | . | . | . | . | L | . | G | . | . | . | . | . | . | . | B.1.143 |
| hCoV-19/India/GBRC21/2020\|EPI ISL 437450\| | . | . | . | . | . | . | . | . | . | . | . | . | L | . | G | . | . | . | . | . | . | . | B.1 |
| hCoV-19/India/781/2020\|EPI ISL 420553\| | . | . | . | . | F | . | . | . | . | . | . | . | L | . | G | . | . | . | . | . | . | . | B.1 |
| hCoV-19/India/777/2020\|EPI ISL 420551\| | . | . | . | . | F | . | . | . | . | . | . | . | L | . | G | . | . | . | . | . | . | . | B.1 |
| hCoV-19/India/773/2020\|EPI ISL 420549\| | . | . | . | . | F | . | . | . | . | . | . | . | L | . | G | . | . | . | . | . | . | . | B.1 |
| hCoV-19/India/772/2020\|EPI ISL 420547\| | . | . | . | . | F | . | . | . | . | . | . | . | L | . | G | . | . | . | . | . | . | . | B.1 |
| hCoV-19/India/770/2020\|EPI ISL 420545\| | . | . | . | . | F | . | . | . | . | . | . | . | L | . | G | . | . | . | . | . | . | . | B.1 |
| hCoV-19/India/1135/2020\|EPI ISL 424362\| | . | . | . | . | F | . | . | . | . | . | . | . | L | . | G | . | . | . | . | . | . | . | B.1 |
| hCoV-19/India/763/2020\|EPI ISL 420543\| | . | . | . | . | F | . | . | . | . | . | . | . | L | . | G | . | . | . | . | . | . | . | B.1 |
| hCoV-19/India/3239/2020\|EPI ISL 424365\| | . | . | . | . | . | . | . | . | . | . | . | . | L | . | G | . | . | . | . | . | . | . | B.1 |
| hCoV-19/India/3118/2020\|EPI ISL 424364\| | . | . | . | . | . | . | . | . | . | . | . | . | L | . | G | . | . | . | . | . | . | . | B.1 |
| hCoV-19/India/NCDC-4874/2020\|EPI ISL 436459\| | . | . | . | . | . | . | . | . | . | . | . | . | L | . | G | H | . | . | . | . | . | . | B.1 |
| hCoV-19/India/GMC-KP1125/2020\|EPI ISL 437626\| | . | . | . | . | . | . | . | . | . | . | . | . | L | . | G | H | . | . | . | . | . | . | B.1 |
| hCoV-19/India/GBRC90/2020\|EPI ISL 447552\| | . | . | . | . | . | . | . | . | . | . | . | . | L | . | G | H | . | . | . | . | . | . | B.1 |
| hCoV-19/India/GBRC49/2020\|EPI ISL 444480\| | . | . | . | . | . | . | . | . | . | . | . | . | L | . | G | H | . | . | . | . | . | . | B.1 |
| hCoV-19/India/GBRC25/2020\|EPI ISL 444456\| | . | . | . | . | . | . | . | . | . | . | . | . | L | . | G | H | . | . | . | . | . | . | B.1 |
| hCoV-19/India/GBRC254b/2020\|EPI ISL 483841\| | . | . | . | . | . | . | . | . | . | . | . | . | L | . | G | H | . | . | . | L | . | . | B.1.113 |
| hCoV-19/India/GBRC254a/2020\|EPI ISL 483840\| | . | . | . | . | . | . | . | . | . | . | . | . | L | . | G | H | . | . | . | L | . | . | B.1.36.8 |
| hCoV-19/India/GBRC91/2020\|EPI ISL 447553\| | . | . | . | . | . | . | . | . | . | . | . | . | L | . | G | H | . | . | . | L | . | . | B.1.36.8 |
| hCoV-19/India/c32/2020\|EPI ISL 420555\| | . | . | . | . | . | . | . | . | . | . | . | . | L | . | G | . | . | . | . | . | K | R | B.1.1 |
| hCoV-19/India/GMC-TC469/2020\|EPI ISL 431117\| | . | . | . | . | . | . | . | . | . | . | . | . | L | . | G | . | . | . | . | . | K | R | B.1.1 |
| hCoV-19/India/NIV-QA-710/2020\|EPI ISL 454565\| | . | . | . | . | . | . | . | . | . | . | . | . | L | . | G | . | . | . | . | . | K | R | B.1.1.101 |
| hCoV-19/India/NIV-65813/2020\|EPI ISL 479565\| | . | . | . | . | . | . | . | . | . | - | . | . | L | . | G | . | . | . | . | . | K | R | B.1.1.306 |
| hCoV-19/India/NIV-64478/2020\|EPI ISL 479555\| | . | . | . | . | . | . | . | . | . | - | . | . | L | . | G | . | . | . | . | . | K | R | B.1.1.306 |
| hCoV-19/India/NIV-20134/2020\|EPI ISL 479513\| | . | . | . | . | . | . | . | . | . | ? | . | . | L | . | G | . | . | . | . | . | K | R | B.1.1.306 |
| hCoV-19/India/1111/2020\|EPI ISL 421666\| | . | I | . | . | . | . | . | . | . | F | . | . | . | . | . | . | . | . | . | . | . | . | B.4 |
| hCoV-19/India/1125/2020\|EPI ISL 421668\| | . | I | . | . | . | . | . | . | . | F | . | . | . | . | . | . | . | . | . | . | . | . | B.4 |
| hCoV-19/India/NCDC-01604/2020\|EPI ISL 435103\| | C | I | . | . | . | . | . | . | I | F | . | . | . | . | . | . | . | . | . | . | . | . | B.4 |
| hCoV-19/India/NCDC-01444/2020\|EPI ISL 435102\| | C | I | . | . | . | . | . | . | I | F | . | . | . | . | . | . | . | . | . | . | . | . | B.4 |
| hCoV-19/India/1652/2020\|EPI ISL 424363\| | C | I | . | . | . | . | . | . | I | F | . | . | . | . | . | . | . | . | . | . | . | . | B.4 |
| hCoV-19/India/1644/2020\|EPI ISL 421672\| | C | I | . | . | . | . | . | . | I | F | . | . | . | . | . | . | . | . | . | . | . | . | B.4 |
| hCoV-19/India/1621/2020\|EPI ISL 421671\| | C | I | . | . | . | . | . | . | I | F | . | . | . | . | . | . | . | . | . | . | . | . | B.4 |
| hCoV-19/India/1617/2020\|EPI ISL 421670\| | C | I | . | . | . | . | . | . | I | F | . | . | . | . | . | . | . | . | . | . | . | . | B.4 |
| hCoV-19/India/1616/2020\|EPI ISL 421669\| | C | I | . | . | . | . | . | . | I | F | . | . | . | . | . | . | . | . | . | . | . | . | B.4 |
| hCoV-19/India/1063/2020\|EPI ISL 424361\| | C | I | . | . | . | . | . | . | I | F | . | . | . | . | . | . | . | . | . | . | . | . | B.4 |
| hCoV-19/India/1115/2020\|EPI ISL 421667\| | C | I | . | . | . | . | . | . | I | F | . | . | . | . | . | . | . | . | . | . | . | . | B.4 |
| hCoV-19/India/1104/2020\|EPI ISL 421665\| | C | I | . | . | . | . | . | . | I | F | . | . | . | . | . | . | . | . | . | . | . | . | B.4 |
| hCoV-19/India/1100/2020\|EPI ISL 421664\| | C | I | . | . | . | . | . | . | I | F | . | . | . | . | . | . | . | . | . | . | . | . | B.4 |
| hCoV-19/India/1093/2020\|EPI ISL 421663\| | C | I | . | . | . | . | . | . | I | F | . | . | . | . | . | . | . | . | . | . | . | . | B.4 |
| hCoV-19/India/1073/2020\|EPI ISL 421662\| | C | I | . | . | . | . | . | . | I | F | . | . | . | . | . | . | . | . | . | . | . | . | B.4 |
| hCoV-19/India/GBRC24a/2020\|EPI ISL 437453\| | . | . | . | . | . | . | K | . | . | . | V | . | . | . | . | . | . | . | L | . | . | . | B.6 |
| hCoV-19/India/GBRC24b/2020\|EPI ISL 437454\| | . | . | . | . | . | . | K | . | . | F | V | G | L | . | . | H | . | . | L | . | . | . | B.6 |
| hCoV-19/India/NCDC-3264/2020\|EPI ISL 436432\| | . | . | . | . | . | . | K | . | . | F | V | . | . | . | . | . | . | . | L | . | . | . | B.6 |
| hCoV-19/India/NCDC-02252/2020\|EPI ISL 435097\| | . | . | . | . | . | . | K | . | . | F | V | . | . | . | . | . | . | . | L | . | . | . | B.6 |
| hCoV-19/India/GMC-RR1191/2020\|EPI ISL 438138\| | . | . | . | . | . | . | K | . | . | F | V | . | . | . | . | . | . | . | L | . | . | . | B.6 |
| hCoV-19/India/GMC-RK1090/2020\|EPI ISL 438139\| | . | . | . | . | . | . | K | . | . | F | V | . | . | . | . | . | . | . | L | . | . | . | B.6 |
| MCL-20-H-2349 F1.4 RNA 2137 | . | . | S | S | . | R | K | T | . | F | V | . | . | V | . | . | . | . | L | . | . | . | B.6.6 |
| MCL-20-H-2347 F2.2 RNA 2135 | . | . | S | S | . | R | K | T | . | F | V | . | . | V | . | . | . | . | L | . | . | . | B.6.6 |
| MCL-20-H-2345 F3.1 RNA 2133 | . | . | S | S | . | R | K | T | . | F | V | . | . | V | . | . | . | . | L | . | . | . | B.6.6 |
| MCL-20-H-2340 F4W RNA 2128 | . | . | S | S | . | R | K | T | . | F | V | . | . | V | . | . | . | . | L | . | . | . | B.6.6 |
| MCL-20-H-2339_F2 RNA 2127 | . | . | S | S | . | R | K | T | . | F | V | . | . | V | . | . | . | . | L | . | . | . | B.6.6 |
| MCL-20-H-2346_F3_RNA 2134 | . | . | S | S | . | R | K | T | . | F | V | . | . | V | . | . | . | . | L | . | . | . | B.6.6 |
| MCL-20-H-2343_F4.1 RNA 2131 | . | . | S | S | . | R | K | T | . | F | V | . | . | V | . | . | . | . | L | . | . | . | B.6.6 |
| MCL-20-H-2337_F2W_RNA 2125 | . | . | S | S | . | R | K | T | . | F | V | . | . | V | . | . | . | . | L | . | . | . | B.6.6 |
