# Supplementary material for: Molecular epidemiology of a familial cluster of SARS-CoV-2 infection during lockdown period in Sant Kabir Nagar, Uttar Pradesh, India
Source: Epidemiol Infect. 2021 Aug 25;149:e200. doi: 10.1017/S0950268821001989 (PMC8438426; doi:10.1017/S0950268821001989)
Supplement: Supplementary file 1 [file hygsup.zip › S0950268821001989sup002.docx]

Technical Appendix Table 2: *Per cent nucleotide and amino acid similarity of the different genes of Sant Kabir Nagar, UP SARS-CoV-2 sequences retrieved in this study along with representative GISAID SARS-CoV-2 sequences with respect to the NC_045512.2_Sars-CoV_2_isolate_Wuhan-Hu-1(Abbreviation: PNS- percent nucleotide similarity; PAS-percent amino acid similarity.*

| **NC_045512.2_SARS-Cov-2_isolate_Wuhan-Hu-1** | **ORF1ab** | | **S** | | **ORF3a** | | **E** | | **M** | | **ORF6** | | **ORF7a** | | **ORF8** | | **N** | | **ORF10** | |
| --- | --- | --- | --- | --- | --- | --- | --- | --- | --- | --- | --- | --- | --- | --- | --- | --- | --- | --- | --- | --- |
|  | **PNS** | **PAS** | **PNS** | **PAS** | **PNS** | **PAS** | **PNS** | **PAS** | **PNS** | **PAS** | **PNS** | **PAS** | **PNS** | **PAS** | **PNS** | **PAS** | **PNS** | **PAS** | **PNS** | **PAS** |
| hCoV-19/India/NIV-QA-710/2020\|EPI_ISL_454565\| | 100.0 | 100.0 | 100.0 | 99.9 | 100.0 | 100.0 | 100.0 | 100.0 | 100.0 | 100.0 | 100.0 | 100.0 | 100.0 | 100.0 | 100.0 | 100.0 | 99.8 | 99.5 | 100.0 | 100.0 |
| hCoV-19/India/NIV-65813/2020\|EPI_ISL_479565\| | 100.0 | 99.9 | 100.0 | 99.9 | 100.0 | 100.0 | 100.0 | 100.0 | 100.0 | 100.0 | 100.0 | 100.0 | 100.0 | 100.0 | 100.0 | 100.0 | 99.8 | 99.5 | 100.0 | 100.0 |
| hCoV-19/India/NIV-64478/2020\|EPI_ISL_479555\| | 100.0 | 100.0 | 100.0 | 99.9 | 100.0 | 100.0 | 100.0 | 100.0 | 100.0 | 100.0 | 100.0 | 100.0 | 100.0 | 100.0 | 100.0 | 100.0 | 99.8 | 99.5 | 100.0 | 100.0 |
| hCoV-19/India/NIV-20134/2020\|EPI_ISL_479513\| | 100.0 | 100.0 | 100.0 | 99.9 | 100.0 | 100.0 | 100.0 | 100.0 | 100.0 | 100.0 | 100.0 | 100.0 | 100.0 | 100.0 | 100.0 | 100.0 | 99.8 | 99.5 | 100.0 | 100.0 |
| hCoV-19/India/NCDC-4874/2020\|EPI_ISL_436459\| | 100.0 | 100.0 | 100.0 | 99.9 | 99.8 | 99.3 | 100.0 | 100.0 | 99.9 | 100.0 | 100.0 | 100.0 | 100.0 | 100.0 | 100.0 | 100.0 | 100.0 | 100.0 | 100.0 | 100.0 |
| hCoV-19/India/NCDC-3264/2020\|EPI_ISL_436432\| | 100.0 | 100.0 | 100.0 | 100.0 | 99.9 | 99.6 | 100.0 | 100.0 | 100.0 | 100.0 | 100.0 | 100.0 | 100.0 | 100.0 | 100.0 | 100.0 | 99.9 | 99.8 | 100.0 | 100.0 |
| hCoV-19/India/NCDC-02252/2020\|EPI_ISL_435097\| | 100.0 | 100.0 | 100.0 | 100.0 | 100.0 | 100.0 | 100.0 | 100.0 | 100.0 | 100.0 | 100.0 | 100.0 | 100.0 | 100.0 | 100.0 | 100.0 | 99.9 | 99.8 | 100.0 | 100.0 |
| hCoV-19/India/NCDC-01604/2020\|EPI_ISL_435103\| | 100.0 | 99.9 | 100.0 | 100.0 | 99.9 | 100.0 | 100.0 | 100.0 | 100.0 | 100.0 | 100.0 | 100.0 | 100.0 | 100.0 | 100.0 | 100.0 | 100.0 | 100.0 | 100.0 | 100.0 |
| hCoV-19/India/NCDC-01457/2020\|EPI_ISL_435108\| | 100.0 | 99.9 | 100.0 | 99.9 | 99.9 | 99.6 | 100.0 | 100.0 | 100.0 | 100.0 | 100.0 | 100.0 | 100.0 | 100.0 | 100.0 | 100.0 | 100.0 | 100.0 | 100.0 | 100.0 |
| hCoV-19/India/NCDC-01444/2020\|EPI_ISL_435102\| | 100.0 | 99.9 | 100.0 | 100.0 | 99.9 | 100.0 | 100.0 | 100.0 | 99.9 | 100.0 | 100.0 | 100.0 | 100.0 | 100.0 | 100.0 | 100.0 | 100.0 | 100.0 | 100.0 | 100.0 |
| hCoV-19/India/GMC-TC469/2020\|EPI_ISL_431117\| | 100.0 | 100.0 | 100.0 | 99.9 | 100.0 | 100.0 | 100.0 | 100.0 | 100.0 | 100.0 | 100.0 | 100.0 | 100.0 | 100.0 | 100.0 | 100.0 | 99.8 | 99.5 | 100.0 | 100.0 |
| hCoV-19/India/GMC-RR1191/2020\|EPI_ISL_438138\| | 100.0 | 100.0 | 100.0 | 100.0 | 100.0 | 100.0 | 100.0 | 100.0 | 100.0 | 100.0 | 100.0 | 100.0 | 100.0 | 100.0 | 100.0 | 100.0 | 99.9 | 99.8 | 100.0 | 100.0 |
| hCoV-19/India/GMC-RK1090/2020\|EPI_ISL_438139\| | 100.0 | 100.0 | 100.0 | 100.0 | 100.0 | 100.0 | 100.0 | 100.0 | 100.0 | 100.0 | 100.0 | 100.0 | 100.0 | 100.0 | 100.0 | 100.0 | 99.9 | 99.8 | 100.0 | 100.0 |
| hCoV-19/India/GMC-RK100/2020\|EPI_ISL_431101\| | 100.0 | 100.0 | 100.0 | 99.9 | 100.0 | 100.0 | 100.0 | 100.0 | 100.0 | 100.0 | 100.0 | 100.0 | 100.0 | 100.0 | 100.0 | 100.0 | 99.9 | 99.8 | 100.0 | 100.0 |
| hCoV-19/India/GMC-KP1125/2020\|EPI_ISL_437626\| | 100.0 | 100.0 | 100.0 | 99.9 | 99.9 | 99.6 | 100.0 | 100.0 | 100.0 | 100.0 | 100.0 | 100.0 | 100.0 | 100.0 | 100.0 | 100.0 | 100.0 | 100.0 | 100.0 | 100.0 |
| hCoV-19/India/GBRC91/2020\|EPI_ISL_447553\| | 100.0 | 100.0 | 100.0 | 99.9 | 99.9 | 99.6 | 100.0 | 100.0 | 99.9 | 100.0 | 100.0 | 100.0 | 100.0 | 100.0 | 100.0 | 100.0 | 99.9 | 99.8 | 100.0 | 100.0 |
| hCoV-19/India/GBRC90/2020\|EPI_ISL_447552\| | 100.0 | 100.0 | 100.0 | 99.9 | 99.8 | 99.3 | 100.0 | 100.0 | 99.9 | 100.0 | 100.0 | 100.0 | 100.0 | 100.0 | 100.0 | 100.0 | 99.9 | 100.0 | 100.0 | 100.0 |
| hCoV-19/India/GBRC9/2020\|EPI_ISL_435056\| | 100.0 | 100.0 | 100.0 | 99.9 | 100.0 | 100.0 | 100.0 | 100.0 | 100.0 | 100.0 | 100.0 | 100.0 | 100.0 | 100.0 | 100.0 | 100.0 | 100.0 | 100.0 | 100.0 | 100.0 |
| hCoV-19/India/GBRC49/2020\|EPI_ISL_444480\| | 100.0 | 100.0 | 100.0 | 99.9 | 99.9 | 99.6 | 100.0 | 100.0 | 99.9 | 100.0 | 100.0 | 100.0 | 99.7 | 100.0 | 100.0 | 100.0 | 99.8 | 100.0 | 100.0 | 100.0 |
| hCoV-19/India/GBRC254b/2020\|EPI_ISL_483841\| | 100.0 | 100.0 | 99.9 | 99.8 | 99.8 | 99.3 | 100.0 | 100.0 | 99.9 | 100.0 | 100.0 | 100.0 | 100.0 | 100.0 | 99.7 | 99.2 | 99.9 | 99.8 | 100.0 | 100.0 |
| hCoV-19/India/GBRC254a/2020\|EPI_ISL_483840\| | 100.0 | 100.0 | 99.9 | 99.8 | 99.9 | 99.6 | 100.0 | 100.0 | 99.9 | 100.0 | 100.0 | 100.0 | 100.0 | 100.0 | 100.0 | 100.0 | 99.9 | 99.8 | 100.0 | 100.0 |
| hCoV-19/India/GBRC25/2020\|EPI_ISL_444456\| | 100.0 | 100.0 | 100.0 | 99.9 | 99.9 | 99.6 | 100.0 | 100.0 | 99.9 | 100.0 | 100.0 | 100.0 | 100.0 | 100.0 | 100.0 | 100.0 | 100.0 | 100.0 | 100.0 | 100.0 |
| hCoV-19/India/GBRC24b/2020\|EPI_ISL_437454\| | 100.0 | 99.9 | 99.9 | 99.9 | 99.8 | 99.3 | 100.0 | 100.0 | 100.0 | 100.0 | 100.0 | 100.0 | 100.0 | 100.0 | 100.0 | 100.0 | 99.9 | 99.8 | 100.0 | 100.0 |
| hCoV-19/India/GBRC24a/2020\|EPI_ISL_437453\| | 100.0 | 99.9 | 100.0 | 100.0 | 100.0 | 100.0 | 100.0 | 100.0 | 100.0 | 100.0 | 100.0 | 100.0 | 100.0 | 100.0 | 100.0 | 100.0 | 99.9 | 99.8 | 100.0 | 100.0 |
| hCoV-19/India/GBRC21/2020\|EPI_ISL_437450\| | 100.0 | 100.0 | 100.0 | 99.8 | 100.0 | 100.0 | 100.0 | 100.0 | 100.0 | 100.0 | 100.0 | 100.0 | 100.0 | 100.0 | 100.0 | 100.0 | 100.0 | 100.0 | 100.0 | 100.0 |
| hCoV-19/India/GBRC113/2020\|EPI_ISL_451158\| | 100.0 | 100.0 | 100.0 | 99.9 | 100.0 | 100.0 | 100.0 | 100.0 | 100.0 | 100.0 | 100.0 | 100.0 | 100.0 | 100.0 | 100.0 | 100.0 | 100.0 | 100.0 | 100.0 | 100.0 |
| hCoV-19/India/c32/2020\|EPI_ISL_420555\| | 100.0 | 100.0 | 100.0 | 99.9 | 100.0 | 100.0 | 100.0 | 100.0 | 100.0 | 100.0 | 100.0 | 100.0 | 100.0 | 100.0 | 100.0 | 100.0 | 99.8 | 99.5 | 100.0 | 100.0 |
| hCoV-19/India/781/2020\|EPI_ISL_420553\| | 100.0 | 100.0 | 100.0 | 99.9 | 100.0 | 100.0 | 100.0 | 100.0 | 100.0 | 100.0 | 100.0 | 100.0 | 100.0 | 100.0 | 100.0 | 100.0 | 100.0 | 100.0 | 100.0 | 100.0 |
| hCoV-19/India/777/2020\|EPI_ISL_420551\| | 100.0 | 100.0 | 100.0 | 99.9 | 100.0 | 100.0 | 100.0 | 100.0 | 100.0 | 100.0 | 100.0 | 100.0 | 100.0 | 100.0 | 100.0 | 100.0 | 100.0 | 100.0 | 100.0 | 100.0 |
| hCoV-19/India/773/2020\|EPI_ISL_420549\| | 100.0 | 100.0 | 100.0 | 99.9 | 100.0 | 100.0 | 100.0 | 100.0 | 100.0 | 100.0 | 100.0 | 100.0 | 100.0 | 100.0 | 100.0 | 100.0 | 100.0 | 100.0 | 100.0 | 100.0 |
| hCoV-19/India/772/2020\|EPI_ISL_420547\| | 100.0 | 100.0 | 100.0 | 99.9 | 100.0 | 100.0 | 100.0 | 100.0 | 100.0 | 100.0 | 100.0 | 100.0 | 100.0 | 100.0 | 100.0 | 100.0 | 100.0 | 100.0 | 100.0 | 100.0 |
| hCoV-19/India/770/2020\|EPI_ISL_420545\| | 100.0 | 100.0 | 100.0 | 99.9 | 100.0 | 100.0 | 100.0 | 100.0 | 100.0 | 100.0 | 100.0 | 100.0 | 100.0 | 100.0 | 100.0 | 100.0 | 100.0 | 100.0 | 100.0 | 100.0 |
| hCoV-19/India/763/2020\|EPI_ISL_420543\| | 100.0 | 100.0 | 100.0 | 99.9 | 100.0 | 100.0 | 100.0 | 100.0 | 100.0 | 100.0 | 100.0 | 100.0 | 100.0 | 100.0 | 100.0 | 100.0 | 100.0 | 100.0 | 100.0 | 100.0 |
| hCoV-19/India/3239/2020\|EPI_ISL_424365\| | 100.0 | 100.0 | 100.0 | 99.9 | 100.0 | 100.0 | 100.0 | 100.0 | 99.9 | 99.6 | 100.0 | 100.0 | 100.0 | 100.0 | 100.0 | 100.0 | 100.0 | 100.0 | 100.0 | 100.0 |
| hCoV-19/India/3118/2020\|EPI_ISL_424364\| | 100.0 | 100.0 | 100.0 | 99.9 | 100.0 | 100.0 | 100.0 | 100.0 | 99.9 | 99.6 | 100.0 | 100.0 | 100.0 | 100.0 | 100.0 | 100.0 | 100.0 | 100.0 | 100.0 | 100.0 |
| hCoV-19/India/1652/2020\|EPI_ISL_424363\| | 100.0 | 99.9 | 100.0 | 100.0 | 100.0 | 100.0 | 100.0 | 100.0 | 100.0 | 100.0 | 100.0 | 100.0 | 100.0 | 100.0 | 100.0 | 100.0 | 99.9 | 100.0 | 100.0 | 100.0 |
| hCoV-19/India/1644/2020\|EPI_ISL_421672\| | 100.0 | 99.9 | 100.0 | 100.0 | 100.0 | 100.0 | 100.0 | 100.0 | 100.0 | 100.0 | 100.0 | 100.0 | 100.0 | 100.0 | 100.0 | 100.0 | 99.9 | 100.0 | 100.0 | 100.0 |
| hCoV-19/India/1621/2020\|EPI_ISL_421671\| | 100.0 | 99.9 | 100.0 | 99.9 | 100.0 | 100.0 | 100.0 | 100.0 | 100.0 | 100.0 | 100.0 | 100.0 | 100.0 | 100.0 | 100.0 | 100.0 | 99.9 | 100.0 | 100.0 | 100.0 |
| hCoV-19/India/1617/2020\|EPI_ISL_421670\| | 100.0 | 99.9 | 100.0 | 100.0 | 100.0 | 100.0 | 100.0 | 100.0 | 100.0 | 100.0 | 100.0 | 100.0 | 100.0 | 100.0 | 99.4 | 98.4 | 99.8 | 99.8 | 100.0 | 100.0 |
| hCoV-19/India/1616/2020\|EPI_ISL_421669\| | 100.0 | 99.9 | 100.0 | 100.0 | 100.0 | 100.0 | 100.0 | 100.0 | 100.0 | 100.0 | 100.0 | 100.0 | 100.0 | 100.0 | 100.0 | 100.0 | 99.9 | 100.0 | 100.0 | 100.0 |
| hCoV-19/India/1-31/2020\|EPI_ISL_413523\| | 100.0 | 100.0 | 100.0 | 99.9 | 100.0 | 100.0 | 100.0 | 100.0 | 100.0 | 100.0 | 100.0 | 100.0 | 100.0 | 100.0 | 99.7 | 99.2 | 100.0 | 100.0 | 100.0 | 100.0 |
| hCoV-19/India/1-27/2020\|EPI_ISL_413522\| | 100.0 | 100.0 | 100.0 | 99.9 | 100.0 | 100.0 | 100.0 | 100.0 | 100.0 | 100.0 | 100.0 | 100.0 | 100.0 | 100.0 | 100.0 | 100.0 | 100.0 | 100.0 | 100.0 | 100.0 |
| hCoV-19/India/1135/2020\|EPI_ISL_424362\| | 100.0 | 100.0 | 99.9 | 99.8 | 100.0 | 100.0 | 100.0 | 100.0 | 100.0 | 100.0 | 100.0 | 100.0 | 100.0 | 100.0 | 100.0 | 100.0 | 100.0 | 100.0 | 100.0 | 100.0 |
| hCoV-19/India/1125/2020\|EPI_ISL_421668\| | 100.0 | 100.0 | 100.0 | 100.0 | 99.9 | 99.6 | 100.0 | 100.0 | 100.0 | 100.0 | 99.5 | 100.0 | 100.0 | 100.0 | 99.7 | 99.2 | 99.9 | 100.0 | 100.0 | 100.0 |
| hCoV-19/India/1115/2020\|EPI_ISL_421667\| | 100.0 | 99.9 | 100.0 | 100.0 | 100.0 | 100.0 | 100.0 | 100.0 | 100.0 | 100.0 | 100.0 | 100.0 | 99.7 | 99.2 | 100.0 | 100.0 | 99.9 | 100.0 | 100.0 | 100.0 |
| hCoV-19/India/1111/2020\|EPI_ISL_421666\| | 100.0 | 100.0 | 100.0 | 100.0 | 100.0 | 100.0 | 100.0 | 100.0 | 100.0 | 100.0 | 100.0 | 100.0 | 100.0 | 100.0 | 100.0 | 100.0 | 99.9 | 100.0 | 100.0 | 100.0 |
| hCoV-19/India/1104/2020\|EPI_ISL_421665\| | 100.0 | 99.9 | 100.0 | 100.0 | 99.9 | 100.0 | 100.0 | 100.0 | 100.0 | 100.0 | 100.0 | 100.0 | 100.0 | 100.0 | 100.0 | 100.0 | 99.9 | 100.0 | 100.0 | 100.0 |
| hCoV-19/India/1100/2020\|EPI_ISL_421664\| | 100.0 | 99.9 | 100.0 | 100.0 | 100.0 | 100.0 | 100.0 | 100.0 | 100.0 | 100.0 | 100.0 | 100.0 | 99.7 | 99.2 | 100.0 | 100.0 | 99.9 | 100.0 | 100.0 | 100.0 |
| hCoV-19/India/1093/2020\|EPI_ISL_421663\| | 100.0 | 99.9 | 100.0 | 100.0 | 99.8 | 99.3 | 100.0 | 100.0 | 100.0 | 100.0 | 100.0 | 100.0 | 99.7 | 99.2 | 100.0 | 100.0 | 99.9 | 100.0 | 100.0 | 100.0 |
| hCoV-19/India/1073/2020\|EPI_ISL_421662\| | 100.0 | 99.9 | 100.0 | 100.0 | 100.0 | 100.0 | 100.0 | 100.0 | 100.0 | 100.0 | 100.0 | 100.0 | 99.7 | 99.2 | 100.0 | 100.0 | 99.9 | 100.0 | 100.0 | 100.0 |
| MCL-20-H-2349 F1.4 RNA 2137 | 100.0 | 99.9 | 100.0 | 100.0 | 100.0 | 100.0 | 100.0 | 100.0 | 100.0 | 100.0 | 100.0 | 100.0 | 100.0 | 100.0 | 99.7 | 100.0 | 99.9 | 99.8 | 100.0 | 100.0 |
| MCL-20-H-2347 F2.2 RNA 2135 | 100.0 | 99.9 | 100.0 | 100.0 | 100.0 | 100.0 | 100.0 | 100.0 | 100.0 | 100.0 | 100.0 | 100.0 | 100.0 | 100.0 | 99.7 | 100.0 | 99.9 | 99.8 | 100.0 | 100.0 |
| MCL-20-H-2345 F3.1 RNA 2133 | 100.0 | 99.9 | 100.0 | 100.0 | 100.0 | 100.0 | 100.0 | 100.0 | 100.0 | 100.0 | 100.0 | 100.0 | 100.0 | 100.0 | 99.7 | 100.0 | 99.9 | 99.8 | 100.0 | 100.0 |
| MCL-20-H-2340 F4W RNA 2128 | 100.0 | 99.9 | 100.0 | 100.0 | 100.0 | 100.0 | 100.0 | 100.0 | 100.0 | 100.0 | 100.0 | 100.0 | 100.0 | 100.0 | 99.7 | 100.0 | 99.9 | 99.8 | 100.0 | 100.0 |
| MCL-20-H-2339_F2 RNA 2127 | 100.0 | 99.9 | 100.0 | 100.0 | 100.0 | 100.0 | 100.0 | 100.0 | 100.0 | 100.0 | 100.0 | 100.0 | 100.0 | 100.0 | 99.7 | 100.0 | 99.9 | 99.8 | 100.0 | 100.0 |
| MCL-20-H-2346_F3_RNA 2134 | 100.0 | 99.9 | 100.0 | 100.0 | 100.0 | 100.0 | 100.0 | 100.0 | 100.0 | 100.0 | 100.0 | 100.0 | 100.0 | 100.0 | 99.7 | 100.0 | 99.9 | 99.8 | 100.0 | 100.0 |
| MCL-20-H-2343_F4.1 RNA 2131 | 100.0 | 99.9 | 100.0 | 100.0 | 100.0 | 100.0 | 100.0 | 100.0 | 100.0 | 100.0 | 100.0 | 100.0 | 100.0 | 100.0 | 99.7 | 100.0 | 99.9 | 99.8 | 100.0 | 100.0 |
| MCL-20-H-2337_F2W_RNA 2125 | 100.0 | 99.9 | 100.0 | 100.0 | 100.0 | 100.0 | 100.0 | 100.0 | 100.0 | 100.0 | 100.0 | 100.0 | 100.0 | 100.0 | 99.7 | 100.0 | 99.9 | 99.8 | 100.0 | 100.0 |
| hCoV-19/India/1063/2020\|EPI_ISL_424361\| | 100.0 | 99.9 | 100.0 | 100.0 | 100.0 | 100.0 | 100.0 | 100.0 | 100.0 | 100.0 | 100.0 | 100.0 | 99.7 | 99.2 | 100.0 | 100.0 | 99.8 | 100.0 | 100.0 | 100.0 |
